# H-NS is the major repressor of *Salmonella* Typhimurium Pef fimbriae expression

**DOI:** 10.1080/21505594.2019.1682752

**Published:** 2019-10-29

**Authors:** Genaro Alejandro Hurtado-Escobar, Olivier Grépinet, Pierre Raymond, Nadia Abed, Philippe Velge, Isabelle Virlogeux-Payant

**Affiliations:** ISP, INRA, Université de Tours, Nouzilly, France

**Keywords:** *Salmonella* Typhimurium, Pef, fimbriae, nucleoproteins, H-NS, Hha

## Abstract

Fimbriae play an important role in adhesion and are therefore essential for the interaction of bacteria with the environments they encounter. Most of them are expressed *in vivo* but not *in vitro*, thus making difficult the full characterization of these fimbriae. Here, we characterized the silencing of plasmid-encoded fimbriae (Pef) expression, encoded by the *pef* operon, in the worldwide pathogen *Salmonella* Typhimurium. We demonstrated that the nucleoid-associated proteins H-NS and Hha, and their respective paralogs StpA and YdgT, negatively regulate at pH 5.1 and pH 7.1 the transcription of the *pef* operon. Two promoters, PpefB and PpefA, direct the transcription of this operon. All the nucleoid-associated proteins silence the PpefB promoter and H-NS also targets the PpefA promoter. While Hha and YdgT are mainly considered as acting primarily through H-NS to modulate gene transcription, our results strongly suggest that Hha and YdgT silence *pef* transcription at acidic pH either by interacting with StpA or independently of H-NS and StpA. We also confirmed the previously described post-transcriptional repression of Pef fimbriae by CsrA titration via the *fim* mRNA and CsrB and CsrC sRNA. Finally, among all these regulators, H-NS clearly appeared as the major repressor of Pef expression. These results open new avenues of research to better characterize the regulation of these bacterial adhesive proteins and to clarify their role in the virulence of pathogens.

## Introduction

Fimbriae are filamentous appendages present on the surface of bacteria which play an important role in the virulence of pathogens. They mediate adhesion to target host cells and to many surfaces encountered by bacteria in their host but also in the environment [1–3]. *Salmonella* Typhimurium genome harbors 13 fimbrial operons: *fim, agf, bcf, sti, sth, lpf, sef, stc, stb, stj, std, stf* and *pef* [4]. All of them have been visualized after heterologous expression in *E. coli* [5] and evidence for their expression by *Salmonella* in animals and/or plants has been observed [6–10]. By contrast, *in vitro, S*. Typhimurium is only able to assemble Type 1 fimbriae encoded by the *fim* operon in standard laboratory growth conditions [11–13]. Altogether, these data are in agreement with a tight regulation of fimbrial expression characterized by a strong *in vitro* repression that can be relieved by the environmental conditions encountered by the bacteria. However, little is known about the physiological environments allowing the expression of fimbriae and the regulatory mechanisms involved.

Plasmid-encoded fimbriae (Pef) of *Salmonella* are thin (2–5 nm diameter) flexible fimbriae belonging to the κ-fimbriae clade characterized by the absence of a tip adhesin [6,14,15]. Pef fimbriae are among the fimbriae whose expression has been observed in animals. Indeed, a low expression of these fimbriae has been identified by flow cytometry after recovery of infected bovine ligated ileal loops [8] and a seroconversion has also been observed after mice or chicken inoculation with *S*. Typhimurium or *S*. Enteritidis respectively [7,16]. Moreover, *pef* mutants of *S*. Typhimurium were shown to be attenuated in their ability to form mature biofilms on a chicken intestinal tissue [17], and also in their colonization of chick, pig, cattle and in a mouse model of long-term systemic persistence [18,19]. Pef fimbriae have also been shown to be necessary for fluid accumulation in the infant mouse [6]. Conversely, Pef fimbriae are not expressed by *Salmonella* in standard laboratory culture conditions, i.e. growth in rich medium at pH 7.0 with or without agitation [7,20–22], except when the genes involved in the biosynthesis of these fimbriae are overexpressed [6,21]. The only culture conditions, known to date, allowing the expression of Pef fimbriae by *Salmonella* are cultures in LB broth buffered at pH 5.1 without agitation [21].

Pef fimbriae biogenesis depends on the *pefBACDorf5orf6* operon, also called *pef* operon, located on the virulence plasmid of *S*. Typhimurium [14]. This operon is also present on the virulence plasmid of *S*. Enteritidis, *S*. Bovismorbificans, *S*. Paratyphi C and *S*. Choleraesuis but *orf5* and *orf6* are not present in all these serotypes. This operon encodes the major Pef fimbriae subunit PefA, the usher protein PefC required for the assembly of the fimbriae and the PefD periplasmic chaperone for PefA. *pefB, orf5* and *orf6* are predicted to encode a regulatory protein and two outer membrane proteins respectively. Two promoters located upstream of *pefB* and *pefA* seem to drive the transcription of this operon [6,23].

Only two papers describe the regulatory mechanisms of Pef fimbriae expression. In 2000, Nicholson and Low [21] described how DNA adenine methylase (Dam) and the leucine-responsive regulatory protein (Lrp) modulate GATC sites methylation upstream of *pefB*, the first open reading frame (ORF) of the *pef* operon, and consequently positively regulate the transcription of this operon. More recently, Sterzenbach et al. [23] characterized how the 5ʹ untranslated region of the *fim* mRNA, encoding Type I fimbriae in *Salmonella*, cooperates with the two small untranslated RNA CsrB and CsrC to prevent Pef fimbriae expression by sequestering the CsrA protein required to stabilize the *pefACDorf5orf6* mRNA. In addition to this latter mechanism, an analysis of the literature led us to postulate that the H-NS, Hha and YdgT nucleoid-associated proteins (NAPs) could also be involved in the repression of Pef fimbriae expression *in vitro*. First, a Δ*hns* mutant was shown to produce more PefA protein than its parental strain [21,22], and then two large transcriptomic studies using *Salmonella* microarrays suggested that the *pef* operon was negatively regulated by the H-NS, Hha and YdgT NAPs [24,25]. Although the three regulators seem thus involved in Pef fimbriae negative regulation, the mechanisms beneath this control remained unknown.

The nucleoprotein H-NS is known to repress the transcription of many genes in Gram-negative enteric bacteria, including virulence genes acquired by horizontal transfer, by directly binding DNA targeted regions in an oligomerized state [26]. In *S*. Typhimurium, almost 10% of the ORFs present on the chromosome or on the virulence plasmid are repressed by H-NS, especially the major virulence genes including most pathogenicity islands [24]. H-NS has a paralog, the nucleoprotein StpA, that is present in *Salmonella* and other enteric bacteria. The StpA and H-NS proteins share 52% identity and display common properties, especially a capacity to bind AT-rich regions found in curved DNA [27]. Moreover, they are able to form heterodimers due to the similarity of their dimerization domains [28]. StpA was shown to regulate 5% of the *Salmonella* genome and most of the StpA regulon is part of the H-NS-dependent genes [29]. Hha and its paralog YdgT belong to the Hha/YmoA family of nucleoproteins whose members are known to interact with other NAPs but do not have a DNA binding domain [30]. These two NAPs exhibit 34% identity and 71% similarity. Hha nucleoprotein has been shown to be a negative regulator for many virulence genes like the enterocyte effacement locus in enterohemorrhagic *E. coli* [31], the *Salmonella* pathogenicity islands 1 (SPI-1) and 2 (SPI-2) [32,33], and the α-hemolysin genes *hlyCABD* in *E. coli* [34]. YdgT seems to act as a support or backup molecule for Hha contributing to the negative regulation of SPI-2 expression in *Salmonella* and the *hly* operon in *E. coli* for example [33,35,36]. To date, the regulatory mechanisms involving Hha and YdgT are not completely understood. Indeed, in 2013, Ali et al. [37] proposed a model where H-NS and Hha nucleoproteins cooperate to repress gene transcription. In this model, H-NS in its oligomerized form binds to the target DNA and interacts with Hha that increases H-NS DNA-binding thanks to positively charged residues on its surface and thus improves H-NS silencing. However, a repression of the transcription of 120 genes by Hha independently of H-NS/StpA has been observed by Solórzano et al. and a direct interaction of Hha with the *rtsA* promoter region has been described [38,39].

Deciphering the regulatory mechanisms of fimbriae expression is important to fully understand the role of these adhesive structures. The objective of our study was thus to demonstrate the repressive effect of H-NS, StpA, Hha and YdgT nucleoproteins on Pef fimbriae expression in *S*. Typhimurium and to evaluate their respective importance in Pef repression compared to the repression exerted by the sequestration of CsrA via the 5ʹUTR of the *fim* mRNA and CsrB/CsrC sRNA.

## Results

### The absence of CsrA sequestration does not allow Pef expression by S. Typhimurium after culture under standing condition at pH 5.1

**Figure 1.**
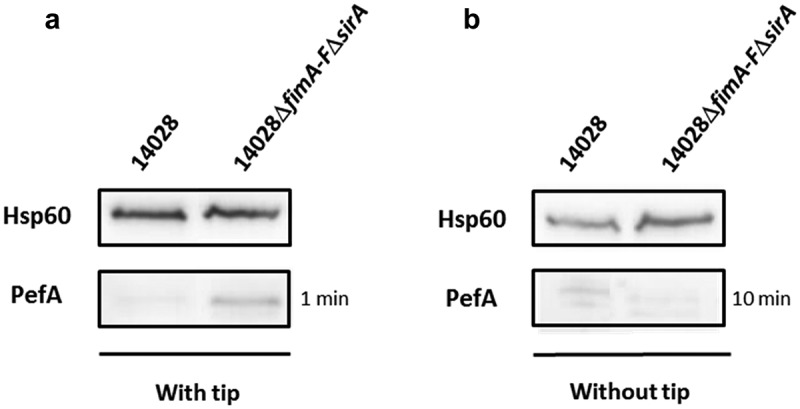
Absence of CsrA sequestration by the 5ʹUTR of *fim* mRNA and CsrB/C allows PefA expression but only in specific culture conditions. Western blot against PefA or Hsp60 (loading control) proteins. *S*. Typhimurium 14028 wild-type and Δ*fimA-F*Δ*sirA* mutants were grown statically at 37°C in TSB-MES pH 5.1 (a) in 5 mL of culture medium put in a 50 mL conical tube containing a sterile 200 μL pipet tip or (b) in 20 mL of culture medium put in a 100 mL flask until stationary phase. For PefA results, the time of membrane exposure necessary to obtain the signal is mentioned.

Nicholson and Low [21] previously observed expression of Pef fimbriae after culture of *S*. Typhimurium in LB broth at pH 5.1 without agitation. More recently, Sterzenbach et al. [23] described the first mechanism of Pef fimbriae repression *in vitro* but in a slightly different culture condition, i.e. in LB buffered to pH 5.5 containing a sterile pipette tip, as a tip was previously shown to enhance Pef fimbriae production *in vitro* [22]. As a first step of our study, we analyzed the importance of this repression mechanism in the culture conditions used by Nicholson and Low as a tip is not an environmental condition encountered by *Salmonella*. As a control, we repeated the experiments of Sterzenbach et al. [23]. For this purpose, we constructed a double Δ*fimA-F*Δ*sirA* mutant of *S*. Typhimurium 14028 strain and performed immunoblots against PefA protein, the major Pef fimbriae subunit. Indeed, in the repression mechanism described by Sterzenbach et al. [23], CsrA is required to stabilize the *pefACDorf5orf6* mRNA. However, *in vitro*, the 5ʹUTR of the *fimAICDHF* transcript cooperates with the CsrB and CsrC sRNA, which are positively regulated by SirA, to antagonize CsrA activity leading to an absence of Pef expression in a wild-type strain (see model Figure 8). Consequently, a double Δ*fimA-F*Δ*sirA* mutant has a marked increase in PefA expression which is related to active CsrA availability in this double mutant that potentiate *pefACDorf5orf6* mRNA translation. Figure 1(a) shows that PefA production was increased in Δ*fimA-F*Δ*sirA* mutants when bacteria were grown statically in conditions similar to that of Sterzenbach et al. [23], i.e. our control conditions. By contrast, we did not observe this increase when bacteria were grown statically in TSB buffered to pH 5.1, the culture conditions used by Nicholson and Low [21] (Figure 1(b)).

These results are in accordance with a role of the 5ʹUTR of the *fimAICDHF* transcript and CsrB and CsrC sRNA in preventing PefA protein production via the sequestration of the CsrA protein. However, they also suggest that this repression depends on the culture conditions used. For the next experiments, as Pef fimbriae have been shown to be expressed statically at pH 5.1 by Nicholson and Low [21], which is a relevant environmental condition encountered by *Salmonella*, we decided to continue our study on the repression of Pef fimbriae expression *in vitro* in this culture condition. We only kept the culture conditions used by Sterzenbach et al. [23] and Nuccio et al. [22] when we tested an impact of CsrA.

### H-NS strongly represses Pef fimbriae expression

**Figure 2.**
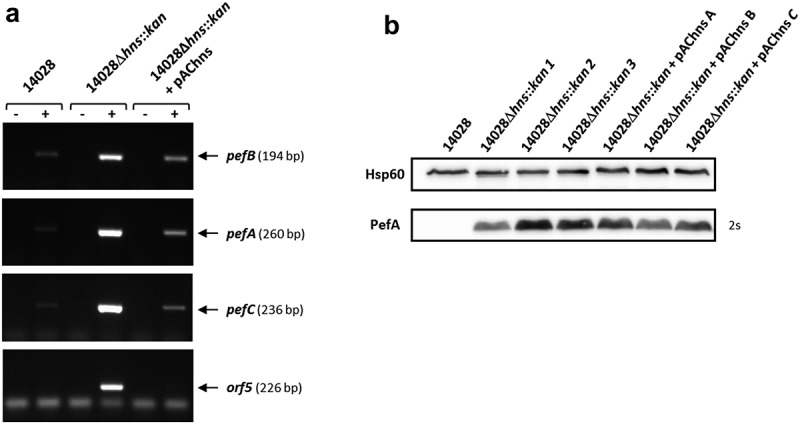
H-NS strongly represses *pef* mRNA level and PefA protein expression in standing cultures at pH 5.1. *S*. Typhimurium 14028 wild-type, Δ*hns::kan* mutants and the complemented strains were grown at 37°C in TSB-MES pH 5.1 under standing conditions. At stationary phase, cells were harvested for RT(reverse transcription)-PCR and Western blot analyses. At least three independent Δ*hns::kan* mutants were tested and the experiment was repeated at least twice. (a) Estimation of *pefB, pefA, pefC* and *orf5* mRNA levels by RT-PCR. PCR were performed on mRNA samples without RT (-) or with RT (+). A representative experiment is presented. (b) Western blot against PefA or Hsp60 (loading control) proteins. For the PefA results, the time of membrane exposure necessary to obtain the signal is mentioned. A typical result obtained with three different Δ*hns::kan* mutants (1 to 3) and three different complemented mutants (a to c) is shown.

The next step of our study was to confirm the repression of Pef fimbriae expression by H-NS. Indeed, as stated in the introduction, several results in the literature suggested a transcriptional repression of the *pef* operon by H-NS [21,24,40]. We first performed RT-PCR experiments on *S*. Typhimurium strains grown statically at 37°C in TSB medium buffered to pH 5.1. We chose to measure the mRNA level of *pefB, pefA, pefC* and *orf5* ORFs which are distributed along the *pef* operon. Moreover, as secondary mutations can occur in Δ*hns* mutants [41], at least 3 independent Δ*hns* mutants were tested in these experiments but also in all the following experiments. A very low level of transcripts was detected in the wild-type strain compared to Δ*hns* mutants for all the ORFs tested. Complementation of the Δ*hns* mutants with pAChns restored the wild-type phenotype (Figure 2(a)). These results demonstrate that H-NS silences *pef* operon transcription.

Immunoblots were then performed to check whether this transcriptional regulation had an impact on PefA protein level. As expected, Western blots revealed a greater amount of PefA protein in Δ*hns* mutants compared to the wild-type strain (Figure 2(b)). It should also be noted that, whatever the samples we tested and the blots we performed, this signal was reproducibly detected after an exposure time of the blots of maximum 2 s for the Δ*hns* mutants while an exposure of about 1 minute was always required to observe PefA expression in Δ*fimA-F*Δ*sirA* mutants (Figure 1). This demonstrates a considerably stronger repression of PefA protein expression by H-NS than by the combined action of the 5ʹUTR of the *fimAICDHF* mRNA and CsrB/CsrC. For unknown reasons and contrary to RT-PCR and β-galactosidase experiments (this paper, see below) and to our previous experiments [42], complementation of the Δ*hns* mutants gave no convincing complementation of PefA protein expression in Western blot experiments (Figure 2(b)). Therefore, all further immunoblot analyses on Δ*hns* mutants in this study, will not include the complemented strain. Altogether, these results demonstrate that H-NS strongly silences Pef fimbriae expression.

### H-NS exerts its repression activity mostly on the PpefB promoter

In order to better characterize the mechanism of *pef* operon regulation by H-NS, we tried to identify the promoter region(s) regulated by this nucleoprotein. So far, two promoters that initiate *pef* operon transcription have been described. Putative −10 and −35 RNA polymerase binding sites have been identified upstream of the *pefB* start codon by Nicholson and Low [21]. These boxes correspond to the transcriptional start site (TSS) recently identified at eight bases downstream of the end of the predicted −10 region and 62 bases upstream of the *pefB* start codon using differential RNA-seq (J. Hinton, personal communication). The second promoter located upstream of *pefA* was recently characterized. The TSS of this promoter is located 76 bases upstream of the *pefA* start codon [23].

**Figure 3.**
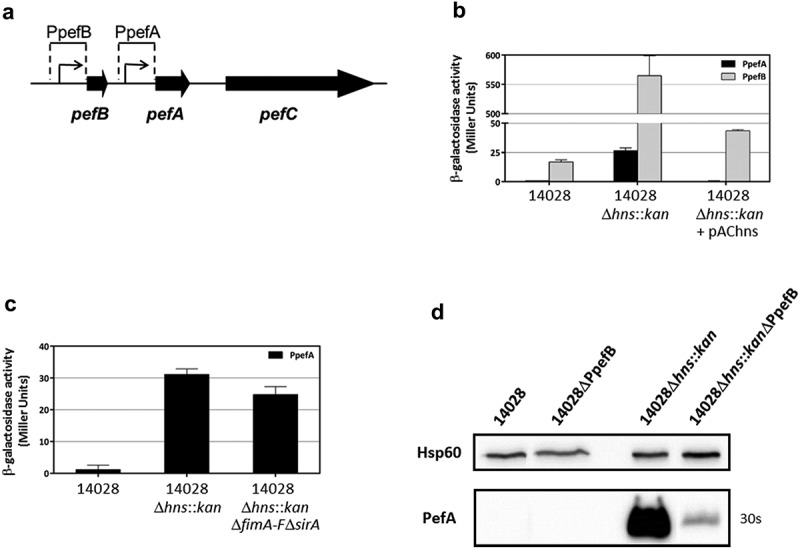
The promoter upstream of *pefB* is the main target of H-NS. (a) Diagram representing the PpefB and PpefA promoters of the *S*. Typhimurium *pef* operon and the DNA fragments cloned upstream of the *lacZ* gene used as a transcriptional reporter. (b and c) β–galactosidase activities expressed by pQF-PpefA or pQF-PpefB transcriptional fusions in *S*. Typhimurium 14028 wild-type strain or its derivatives after growth until stationary phase at 37°C in TSB-MES pH 5.1 under standing conditions without a sterile pipette tip (b) and in presence of a sterile pipette tip (c). Aliquots were collected for measurements of the OD_600nm_ and β–galactosidase activity. β-galactosidase activities are expressed in Miller units. Average values (± standard error of the mean) of activity units were calculated based on at least three independent assays performed in duplicates for each strain. For Δ*hns::kan* strains, several mutants were tested in each assay and results presented are the mean of the data obtained for all these mutants. (d) Western blot against PefA or Hsp60 proteins (loading control) prepared from *S*. Typhimurium strains grown at 37°C until stationary phase in TSB-MES pH 5.1 under standing conditions. For the PefA results, the time of membrane exposure necessary to obtain the signal is mentioned. At least three independent Δ*hns::kan* and Δ*hns::kan*ΔPpefB mutants were tested and a representative result is shown.

Two plasmid-based transcriptional fusions were constructed in the low copy number promoter-probe vector pQF50Cm [43,44] with DNA fragments containing the promoter region upstream of *pefB* (PpefB) or upstream of *pefA* (PpefA) and introduced into the wild-type *S*. Typhimurium 14028 strain. Then Δ*hns::kan* mutation was introduced by transduction in these strains and several mutants harboring each transcriptional fusion were selected for β-galactosidase experiments (Figure 3(a)). Experiments were performed after bacterial culture at 37°C in TSB-MES pH 5.1 under standing conditions. No significant transcription of the *lacZ* reporter gene was detected with the empty vector pQF50Cm in this culture condition (data not shown). Both pQF-PpefB and pQF-PpefA transcriptional fusions exhibited a weak or no β-galactosidase activity in the wild-type strain, 17 ± 1.8 and 0.66 ± 0.13 Miller units respectively. By contrast, a high increase of β-galactosidase activity was observed for the pQF-PpefB fusion in the Δ*hns::kan* mutants (565 ± 34 Miller units) and pAChns plasmid was shown to efficiently restore the wild-type phenotype. These experiments demonstrate that H-NS acts as a strong silencer of the PpefB promoter (Figure 3(b)). Similar results were obtained with the pQF-PpefA::*lacZ* fusion. The β-galactosidase activities of the pQF-PpefA fusion remained low in Δ*hns* mutants (26.5 ± 2.4 Miller Units), but they were nevertheless 40 times higher in Δ*hns* mutants than in the wild-type strain. As CsrA was shown to induce PefA expression by binding to the 5ʹUTR of the *pefACDorf5orf6* transcript [23], we hypothesized that the impact of H-NS on pQF-PpefA fusion could be masked due to the unavailability of active CsrA protein to stabilize *lacZ* mRNA in our experiments. To test this hypothesis, we measured the β-galactosidase activity of this fusion in Δ*hns*Δ*fimA-F*Δ*sirA* mutants. No increase of β-galactosidase activities of the pQF-PpefA fusion was observed in Δ*hns*Δ*fimA-F*Δ*sirA* triple mutants compared to Δ*hns* mutants neither after bacterial growth in TSB-MES pH 5.1 under standing conditions (data not shown) nor after growth in Sterzenbach et al. culture conditions [23] (Figure 3(c)). These results demonstrate that the low β-galactosidase activity of the pQF-PpefA fusion in the Δ*hns* mutants is not due to CsrA sequestration by *fim* operon and CsrB and CsrC sRNA. Together, these results are in favor of a regulation of PpefA promoter by H-NS, even if our experiments were probably not performed in the best environmental conditions for observing this regulation.

To confirm the predominant role of the PpefB promoter in the regulation of PefA expression by H-NS, we deleted the PpefB promoter and performed immunoblot analyses. A decrease of PefA expression was observed in the Δ*hns*ΔPpefB mutants compared to the Δ*hns* mutant strains, demonstrating that the silencing of PefA by H-NS is essentially mediated by the PpefB promoter in our culture conditions (Figure 3(d)). Altogether, these results demonstrate that the main target of H-NS involved in the negative regulation of Pef fimbriae expression is the promoter region located upstream of *pefB* at least when bacteria are cultured in TSB-MES pH 5.1 under standing conditions. However, we have to consider that PpefB is a stronger promoter than PpefA in our culture conditions. We therefore cannot exclude a predominant role of the PpefA promoter in the regulation of Pef fimbriae by H-NS in different culture conditions.

### Hha and YdgT negatively regulate PefA expression but at a lower level than H-NS

**Figure 4.**
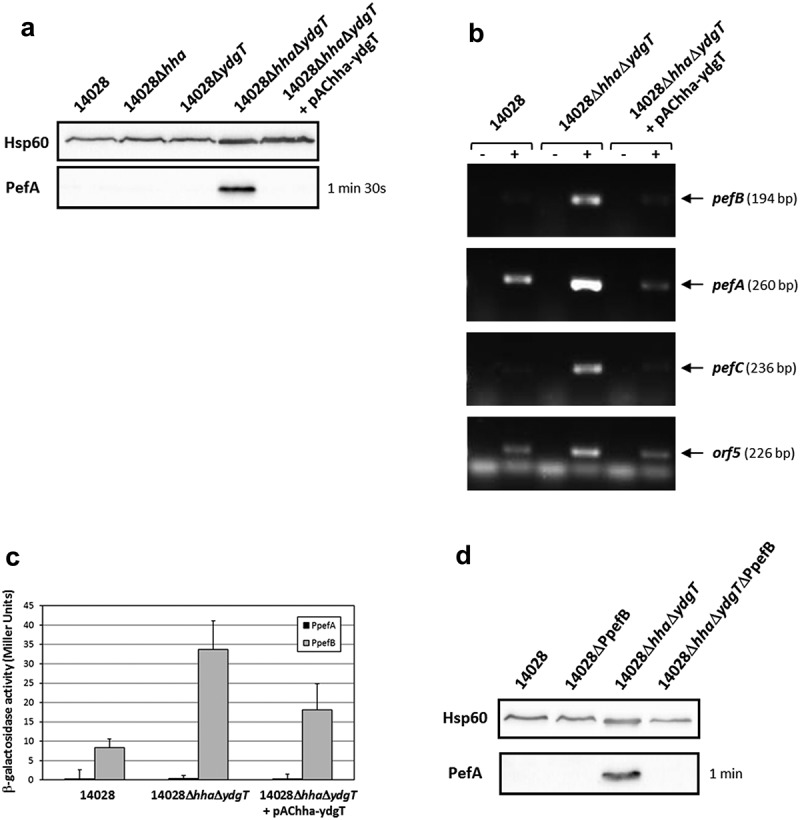
Hha and YdgT negatively regulate PefA expression by acting on the *pefB* promoter. *S*. Typhimurium 14028 and its derivative strains were grown statically at 37°C in TSB-MES pH 5.1. At stationary phase, cells were harvested for Western blot analysis, RT-PCR and β-galactosidase activity measurement. At least three independent assays were performed for each experiment. (a) Western blot against PefA or Hsp60 proteins (loading control) prepared from *S*. Typhimurium wild-type, Δ*hha* and Δ*ydgT* single mutants, Δ*hha*Δ*ydgT* double mutant and its complemented strain. For PefA results, the time of membrane exposure necessary to obtain the signal is mentioned. (b) Estimation of *pefB, pefA, pefC* and *orf5* mRNA levels by RT-PCR. PCR were performed on mRNA samples without RT (-) or with RT (+). (c) β–galactosidase activities expressed by pQF-PpefA and pQF-PpefB transcriptional fusions in *S*. Typhimurium wild-type, Δ*hha*Δ*ydgT* double mutant and its complemented strain. β-galactosidase activities are expressed in Miller units. Average values (± standard error of the mean) of activity are presented. (d) Western blot against PefA or Hsp60 proteins (loading control) prepared from *S*. Typhimurium wild-type, ΔPpefB, Δ*hha*Δ*ydgT* double mutant and the Δ*hha*Δ*ydgT*ΔPpefB triple mutant. For PefA results, the time of membrane exposure necessary to obtain the signal is mentioned.

In addition to H-NS, the nucleoproteins Hha and YdgT have also been suggested to be involved in the repression of Pef fimbriae expression in *S*. Typhimurium [25]. To test this hypothesis, single Δ*hha* or Δ*ydgT* mutants and a double Δ*hha*Δ*ydgT* mutant were constructed. PefA expression was detected only in the double mutant by immunoblot analyses and neither in the wild-type strain nor in the single mutants (Figure 4(a)). The wild-type phenotype was completely restored by complementation of the double Δ*hha*Δ*ydgT* mutant with the pAChha-ydgT plasmid. These results were confirmed by RT-PCR experiments on the double Δ*hha*Δ*ydgT* mutant which exhibited an increase of *pefB, pefA, pefC* and *orf5* mRNA level compared to the wild-type or the complemented strain (Figure 4(b)). These results demonstrate that Hha and YdgT negatively regulate PefA expression and that the simultaneous absence of Hha and YdgT is necessary to relieve this repression.

Determination of the promoter(s) targeted by Hha and YdgT was performed using the transcriptional fusions pQF-PpefA and pQF-PpefB and after bacterial culture at 37°C in TSB-MES pH 5.1 under standing conditions. No transcriptional activity was observed for the PpefA promoter whatever the strain tested, while a low β-galactosidase activity (34 ± 7.4 Miller units), partially complemented by pAChha-ydgT, was detected for the PpefB promoter in the double Δ*hha*Δ*ydgT* mutant (Figure 4(c)). Moreover, deletion of the PpefB promoter in the Δ*hha*Δ*ydgT* mutant abolished the expression of PefA protein as shown by immunoblot analysis (Figure 4(d)). Taken together, these results demonstrate that Hha and YdgT negatively regulate PefA expression by acting, as for H-NS, on the PpefB promoter. However, this repression was much weaker than the one exerted by H-NS as the transcriptional fusion PpefB::*lacZ* displayed a reporter gene expression about 17-fold lower in a double Δ*hha*Δ*ydgT* mutant than in a Δ*hns* mutant. Moreover, acquisition times used for detection of PefA protein on Western blots were always much higher for the double Δ*hha*Δ*ydgT* mutant than for the Δ*hns* mutant (in the minute range versus in the second range respectively).

### Higher PefA expression is observed at pH 7.1 than pH 5.1 in *Δ*hns or *Δ*hha*Δ*ydgT or *Δ*fimA-F*Δ*sirA mutants

**Figure 5.**
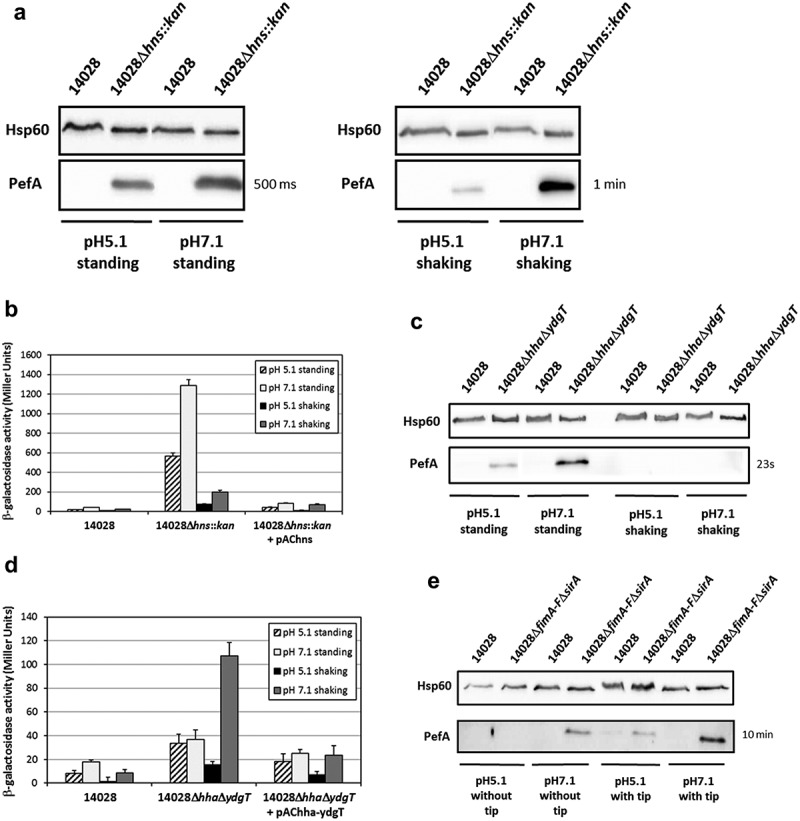
Higher PefA expression at pH 7.1 than pH 5.1 in Δ*hns*, Δ*hha*Δ*ydgT* or Δ*fimA-F*Δ*sirA* mutants. *S*. Typhimurium 14028 and isogenic mutants were grown at 37°C in TSB-MES pH 5.1 or pH 7.1 under standing or shaking conditions. At stationary phase, cells were harvested for Western blot analysis and β-galactosidase activity measurement. (a) Western blot against PefA or Hsp60 proteins (loading control) prepared from wild-type strain and several Δ*hns::kan* mutants. A typical result is presented for each condition tested. For PefA results, the time of membrane exposure necessary to obtain the signal is mentioned. (b) β–galactosidase activities expressed by the transcriptional fusion pQF-PpefB in *S*. Typhimurium 14028 wild-type, Δ*hns::kan* mutant and its complemented strain. Activities are expressed in Miller units. Average values (± standard error of the mean) of activity were calculated based on at least three independent assays. For Δ*hns::kan* strain, several mutants were tested in each assay. (c) Western blot against PefA or Hsp60 proteins (loading control) prepared from wild-type strain and Δ*hha*Δ*ydgT* double mutant. For the PefA results, the time of membrane exposure necessary to obtain the signal is mentioned. (d) β–galactosidase activity expressed by the transcriptional fusion pQF-PpefB in *S*. Typhimurium 14028 wild-type, Δ*hha*Δ*ydgT* double mutant and its complemented strain. Activities are expressed in Miller units. Average values (± standard error of the mean) of activity were calculated based on at least three independent assays. (e) Western blot against PefA or Hsp60 proteins (loading control) prepared from wild-type strain and Δ*fimA-F*Δ*sirA* double mutant. Bacteria were grown with or without a sterile pipette tip added in the culture medium to mimick the conditions used by Sterzenbach et al. who demonstrated the role of *fim* and *sirA* in the regulation of Pef expression [23]. For the PefA results, the time of membrane exposure necessary to obtain the signal is mentioned.

All our previous experiments were performed in culture conditions described in the literature as optimal for the expression of Pef fimbriae, i.e. under standing conditions at pH 5.1 [21]. However, during an experiment designed to study the expression of another target of the Hha/YdgT regulon, we observed an expression of PefA after bacterial culture at pH 7.1 suggesting that an expression of this protein can be detected in culture conditions other than pH 5.1 without agitation. We, thus, decided to study the impact of agitation and pH on *pef* expression in Δ*hns*, Δ*hha*Δ*ydgT* and Δ*fimA-F*Δ*sirA* mutants. Bacteria were grown at pH 5.1 or 7.1 with or without shaking. PefA was detected by immunoblot in Δ*hns* mutants in all the conditions tested but at a higher level in standing cultures than after bacterial growth under shaking conditions (Figure 5(a)). Moreover, the signal detected for PefA was always more intense at pH 7.1 than at pH 5.1. The measure of the transcriptional activity of the PpefB promoter in Δ*hns* mutants in these four culture conditions confirmed these results. Standing cultures allowed a higher expression of *lacZ* reporter gene than shaking cultures (Figure 5(b)). Moreover, in agreement with immunoblot results, the β-galactosidase activities measured for pQF-PpefB fusion (1287 ± 60 Miller units for standing culture and 198 ± 19 Miller units for shaking culture) were always higher at pH 7.1 than at pH 5.1 (565 ± 34 Miller units for standing culture, 72 ± 8 Miller units for shaking culture). The wild-type strain displayed weak β-galactosidase activities, the highest being also those measured in standing conditions at pH 7.1 (40 ± 1.7 Miller units).

Similar experiments were carried out on the double Δ*hha*Δ*ydgT* mutant and the results were generally consistent with those obtained for the Δ*hns* mutants. More precisely, PefA was never detected in shaking cultures even after an immunoblot exposure of several minutes whereas, in standing cultures, PefA was more expressed at pH 7.1 than pH 5.1 (Figure 5(c) and data not shown). Moreover, as previously observed at pH 5.1 in standing cultures, PefA expression was always lower in the Δ*hha*Δ*ydgT* double mutant than in Δ*hns* mutants, confirming the highest repression activity of H-NS compared to Hha and YdgT. β-galactosidase experiments on the PpefB promoter in the Δ*hha*Δ*ydgT* double mutant gave results fully consistent with immunoblot analyses except when bacteria were cultured at pH 7.1 in shaking conditions (Figure 5(d)). In this latter condition, the highest β-galactosidase activity was detected in the Δ*hha*Δ*ydgT* mutant for the pQF-PpefB fusion (107 ± 11 Miller units) whereas PefA protein was not detected in this culture condition (Figure 5(d,c)).

For Δ*fimA-F*Δ*sirA* mutants, the highest expression of PefA protein was detected after bacterial culture under standing conditions at pH 7.1 and with a tip. Moreover, at this pH, PefA was detected even when bacteria were statically cultivated without a tip. This was not the case at pH 5.1 (Figure 5(e)). As for Δ*hha*Δ*ydgT* mutants, no PefA expression was detected after bacterial culture in shaking conditions (data not shown).

In conclusion, these results confirm the importance of the culture conditions for the expression of Pef fimbriae and demonstrate a repression of Pef expression by H-NS in all the culture conditions tested and by Hha/YdgT and CsrA at least in standing culture.

### Deletion of *hha* and *ydgT* in an *hns* mutant represses PefA expression more than in a single *hns* mutant

**Figure 6.**
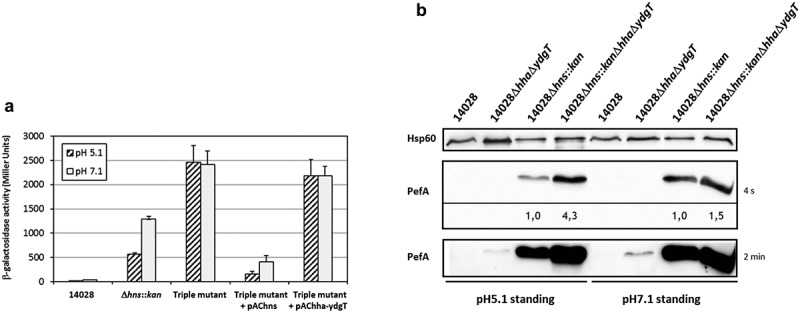
Deletion of *hha* and *ydgT* in a Δ*hns::kan* mutant has a more pronounced effect on PefA expression at pH 5.1 than at pH 7.1. (a) β–galactosidase activity expressed by the transcriptional fusion pQF-PpefB in *S*. Typhimurium Δ*hns::kan*Δ*hha*Δ*ydgT* triple mutant and its complemented strains after growth in TSB-MES pH 5.1 or pH 7.1 at 37°C under standing conditions. In stationary phase, aliquots were collected for measurements of the OD_600nm_ and β–galactosidase activity. β-galactosidase activities are expressed in Miller units. Average values (± standard error of the mean) of activity units were calculated based on at least three independent assays. For Δ*hns::kan* and Δ*hns::kan*Δ*hha*Δ*ydgT* strains, several mutants were tested in each assay. (b) Western blot against PefA or Hsp60 proteins (loading control) prepared from *S*. Typhimurium 14028 wild-type, Δ*hha*Δ*ydgT*, Δ*hns*::kan or Δ*hns::kan*Δ*hha*Δ*ydgT* mutants grown statically at 37°C until stationary phase in TSB-MES pH 5.1 or pH 7.1. Several Δ*hns::kan* mutants and Δ*hns::kan*Δ*hha*Δ*ydgT* triple mutants were tested in each experiment. A typical result is presented for each culture condition tested. For the PefA results, the time of membrane exposure necessary to obtain the signal is mentioned. Relative intensities for PefA bands calculated using normalization with the loading control Hsp60 are noted just under the immunoblot exposed for 4 s and for which no signal saturation was detected.

The nucleoproteins H-NS and, to a lesser extent, Hha-YdgT, are involved in the silencing of Pef fimbriae expression. Subsequently, one of the key questions is whether Hha-YdgT act in synergy with H-NS as it has been shown *in vitro* that Hha can improve H-NS binding to target regions [37]. We reasoned that if Hha and YdgT repress *pef* transcription only through H-NS, then the deletion of *hha* and *ydgT* in a Δ*hns* mutant will have no greater impact on *pef* transcription and PefA expression than the impact of *hns* deletion. We measured the transcriptional activity of the PpefB promoter region in a Δ*hns::kan*Δ*hha*Δ*ydgT* triple mutant grown in standing cultures at pH 5.1 and pH 7.1, which correspond to the culture conditions in which a repression was observed in Δ*hns* and Δ*hha*Δ*ydgT* mutants (Figure 5). At pH 7.1, the activity of the pQF-PpefB transcriptional fusion in the Δ*hns::kan*Δ*hha*Δ*ydgT* triple mutant was about 2-fold higher than in the Δ*hns* mutant (2419 ± 275 versus 1287 ± 60 Miller units) whereas at pH 5.1, the activity in the triple mutant was 4.3-fold higher than in the Δ*hns* mutant (2460 ± 349 versus 565 ± 34 Miller units) (Figures 5(b) and 6(a)). Complementation with pAChns plasmid, contrary to pAChha-ydgT, confirmed that H-NS is the main regulator on this promoter region as the transcriptional activity returned to a very low level in the complemented strain. Then we investigated the expression of PefA in the Δ*hns*Δ*hha*Δ*ydgT* triple mutant grown in similar culture conditions. Expression of PefA was increased 4.3-fold in the triple mutant compared to the Δ*hns* single mutant when bacteria were grown at pH 5.1 whereas both the triple and the Δ*hns* mutant displayed a similar level of PefA expression at pH 7.1 (1.5-fold higher in the triple mutant compared to the Δ*hns* mutant) (Figure 6(b)). These results are consistent with β-galactosidase experiments which showed a greater increase of the PpefB promoter activity in the triple mutant compared to the Δ*hns* mutant at pH 5.1 than pH 7.1 (Figure 6(a)).

Altogether, these results suggest a negative regulation of PefA expression by Hha-YdgT independently of H-NS in the Δ*hns*Δ*hha*Δ*ydgT* triple mutant at pH 5.1. As StpA, the paralog of H-NS, is expressed at high levels in the absence of H-NS [24,40], we tested whether StpA could be responsible for the higher expression of PefA in the triple mutant compared to the Δ*hns* mutant. Unfortunately, we were not able to construct a Δ*hns*Δ*stpA* double mutant. As we succeeded in constructing a Δ*stpA* mutant and in deleting *hns* in a *stpA* mutant complemented with *stpA*, our results strongly suggest that the deletion of both *hns* and *stpA* is lethal for *Salmonella*. Consequently, we cannot rule out a role of StpA in regulating Pef fimbriae expression in an *hns* background and on its involvement with Hha-YdgT in the higher expression of PefA in the Δ*hns*Δ*hha*Δ*ydgT* triple mutant after bacterial culture at pH 5.1.

### StpA represses Pef fimbriae expression by acting on the PpefB promoter

**Figure 7.**
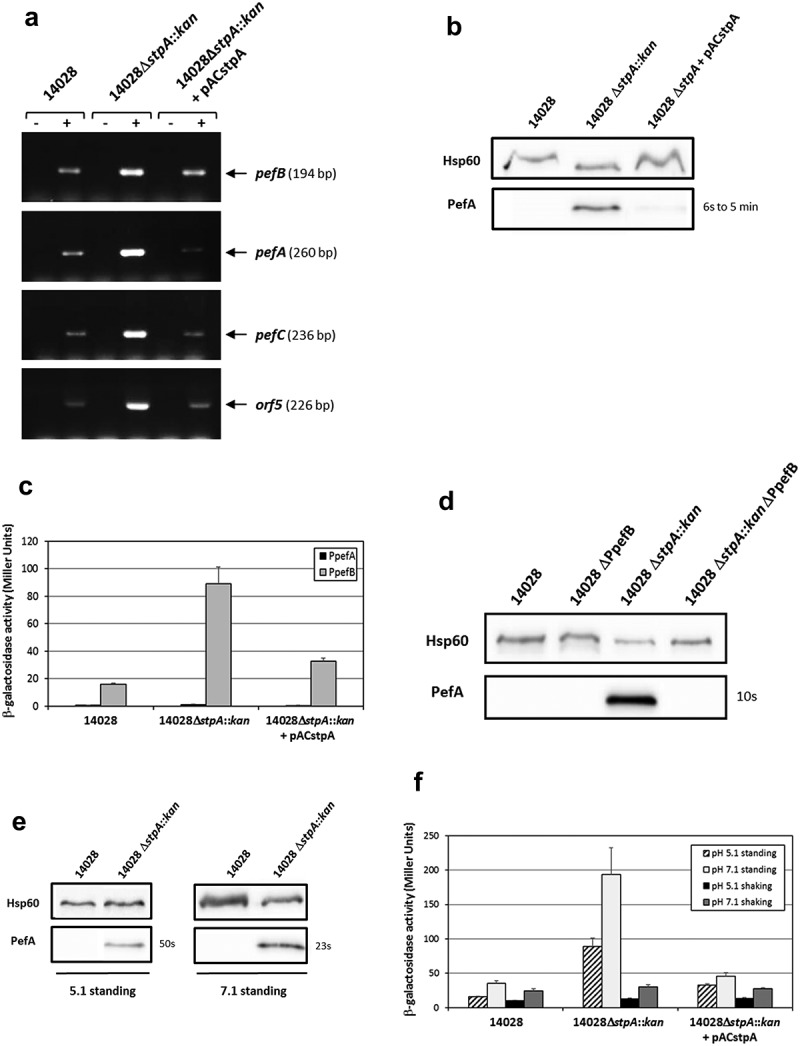
StpA silences PefA expression by acting on the *pefB* promoter. *S*. Typhimurium 14028 and its derivative strains were grown statically at 37°C in TSB-MES pH 5.1 (A to D) or at 37°C in TSB-MES pH 5.1 or pH 7.1 under standing or shaking conditions (e and f). At stationary phase, cells were harvested for RT-PCR, Western blot analysis and β-galactosidase activity measurement. At least three independent assays were performed for each experiment. (a) Estimation of *pefB, pefA, pefC* and *orf5* mRNA levels by RT-PCR. PCR were performed on mRNA samples without RT (-) or with RT (+) and prepared from *S*. Typhimurium wild-type, Δ*stpA::kan* mutant and its complemented strain. (b) Western blot against PefA or Hsp60 proteins (loading control). For the PefA results, the time of membrane exposure necessary to obtain the signal is mentioned. (c) β–galactosidase activities expressed by pQF-PpefA and pQF-PpefB transcriptional fusions in *S*. Typhimurium wild-type, Δ*stpA::kan* mutant and its complemented strain. β-galactosidase activities are expressed in Miller units. Average values (± standard error of the mean) of activity are presented. (d) Western blot against PefA or Hsp60 proteins (loading control) prepared from *S*. Typhimurium wild-type, ΔPpefB, Δ*stpA::kan* mutant and the Δ*stpA::kan*ΔPpefB double mutant. For PefA results, the time of membrane exposure necessary to obtain the signal is mentioned. (e) Western blot against PefA or Hsp60 proteins (loading control) prepared from wild-type strain and Δ*stpA::kan* mutant. For the PefA results, the time of membrane exposure necessary to obtain the signal is mentioned. (f) β–galactosidase activity expressed by the transcriptional fusion pQF-PpefB in *S*. Typhimurium 14028 wild-type, Δ*stpA::kan* mutant and its complemented strain. Activities are expressed in Miller units. Average values (± standard error of the mean) of activity were calculated based on at least three independent assays.

While StpA is highly expressed during early and middle exponential phase, expression of StpA is minimal and low during late stationary phase which corresponds to the culture conditions used in our experiments [29,45]. This is why we initially did not test the H-NS paralog. However, when we tried to explain the higher expression of PefA in the Δ*hns*Δ*hha*Δ*ydgT* triple mutant at pH 5.1, we surprisingly observed that deletion of *stpA* in *S*. Typhimurium 14028 strain led to an increase of PefA expression and decided to study this phenotype. As for Δ*hns* and Δ*hha*Δ*ydgT* mutants, RT-PCR experiments performed on *S*. Typhimurium strains grown statically at 37°C in TSB medium buffered to pH 5.1 showed a higher level of transcripts in the Δ*stpA* mutant than in the wild-type strain for the *pefB, pefA, pefC* and *orf5* ORFs. Complementation of the Δ*stpA* mutant with pACstpA restored the wild-type phenotype (Figure 7(a)). Moreover, a greater amount of PefA protein was detected by immunoblots in Δ*stpA* mutants compared to the wild-type strain or the complemented strain (Figure 7(b)), thus confirming the RT-PCR results. Intriguingly, the exposure times needed to obtain a signal for Δ*stpA* mutants were quite variable depending on the mutant used and on the experiment (between a few seconds up to a few minutes) unlike those obtained for Δ*hns* and Δ*hha*Δ*ydgT* mutants that were highly reproducible. Nevertheless, the exposure times of the blots were always higher than that obtained for Δ*hns* mutants. Then, using the transcriptional fusions pQF-PpefA and pQF-PpefB and by deleting the PpefB promoter in the Δ*stpA* background, we demonstrated that PpefB is the main promoter targeted by StpA, as previously shown for H-NS and Hha/YdgT. Indeed, a moderate β-galactosidase activity (89 ± 12 Miller units), partially complemented by pACstpA, was detected for the PpefB promoter in the Δ*stpA* mutant, while no transcriptional activity was observed for the PpefA promoter whatever the strain tested (Figure 7(c)). Moreover, immunoblot experiments showed that deletion of the PpefB promoter abolished PefA expression in the Δ*stpA* mutant (Figure 7(d)). Altogether, these results demonstrate that StpA silences *pef* operon transcription on the promoter located upstream of *pefB* when bacteria are cultivated at acidic pH 5.1 until late stationary phase.

Finally, we tested the impact of the agitation and pH on *pef* expression in *stpA* mutants. As observed for the double Δ*hha*Δ*ydgT* mutant, (i) PefA expression was detected only in standing culture (Figure 7(e) and data not shown for shaking cultures), (ii) a higher expression of PefA was detected at pH 7.1 than at pH 5.1 (Figure 7(e)) and (iii) a higher β-galactosidase activity for pQF-PpefB fusion (193 ± 39 Miller units for standing culture and 30 ± 3.1 Miller units for shaking culture) was measured at pH 7.1 than at pH 5.1 (89 ± 12 Miller units for standing culture and 13 ± 0.9 Miller units for shaking culture) (Figure 7(f)). However, whatever the culture conditions used, the silencing of Pef by StpA was always lower than that of H-NS.

## Discussion

**Figure 8.**
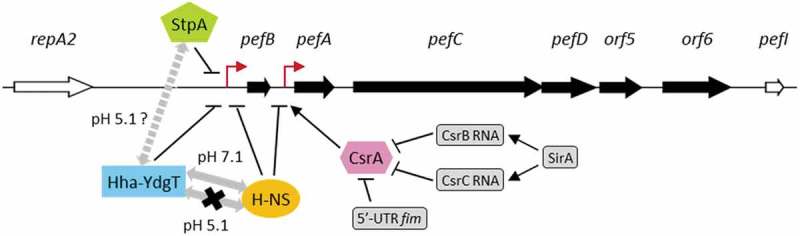
Model for *in vitro pef* regulation in *S*. Typhimurium. The six ORFs belonging to the *pef* operon are represented by black arrows. The two promoters identified upstream of *pefB* and *pefA* are indicated by red broken arrows. The promoter upstream of *pefA* is repressed by H-NS and the RNA binding protein CsrA stabilizes the *pefACDorf5orf6* transcript. The activity of CsrA is antagonized by the 5ʹ-UTR of the *fimA-F* transcript and the two small RNAs named CsrB and CsrC [23]. The promoter upstream of *pefB* is strongly silenced by H-NS and weakly by StpA and Hha-YdgT. The two gray double arrows between H-NS and Hha-YdgT indicate that, at pH 7.1 Hha-YdgT act through H-NS to modulate *pef* expression, contrary to pH 5.1 where Hha-YdgT seem able to negatively regulate *pef* expression independently of H-NS. The single dashed double arrow with a question mark connecting StpA and Hha-YdgT signifies a possible interaction at pH 5.1 between these three NAPs to repress *pef* expression.

Fimbrial expression *in vivo* but not *in vitro* suggests a fine-tuned regulation according to the environment encountered by bacteria allowing them to express these complex structures at their surface only when required and thus to optimize virulence against fitness costs. In *Salmonella*, several genes/proteins have been implicated in the repression of fimbrial expression but the mechanisms of regulation remain unknown [21,46] except in the case of the post-transcriptional control of Pef fimbriae expression via CsrA protein [23]. Analysis of published high throughput transcriptomic data suggested that at least eight of the thirteen fimbrial operons present in the genome of *S*. Typhimurium, including *pef* operon, were negatively regulated by H-NS [24,40]. On the other hand, the *pef* operon is one of the few fimbrial operons which seemed regulated by Hha-YdgT [25]. Our results demonstrate that H-NS strongly silences the *pef* operon transcription at both the PpefB and the PpefA promoters, the two promoters of the *pef* operon currently identified. In our culture conditions, i.e. bacterial culture at pH 5.1 and in standing conditions, the main impact occurs on the transcription initiated at the PpefB promoter. The environmental conditions and the regulatory proteins favoring the transcription from the PpefA promoter are currently unknown. We also show that the PpefB promoter, but not the PpefA promoter, is silenced by Hha-YdgT and StpA, the paralog of H-NS, but these nucleoproteins affect *pef* operon transcription less than H-NS. The use of a double Δ*hha*Δ*ydgT* mutant was necessary to obtain this phenotype, which is consistent with the already described redundancy of these proteins [25,36,47]. Concerning StpA, our results are the first demonstration of StpA gene silencing after bacterial growth in late stationary phase, i.e. a growth phase where StpA is expressed at a low level in *Salmonella* but also in other bacteria [45,48,49]. Indeed, Lucchini et al. [29] in *Salmonella*, but also Müller et al. [50] in *E. coli* for example, did not observe any altered gene expression in a *stpA* mutant compared to the wild-type strain in late stationary phase using DNA arrays. Our results cannot be attributed to a reduced expression of H-NS in the *stpA* mutant as no significant StpA-dependent changes in *hns* expression was observed at any *S*. Typhimurium growth time point [29] in accordance with other transcriptomic studies in *Shigella flexneri* or *E. coli* [48,50]. *pef* ORFs seem not to be the only fimbriae related genes regulated by StpA as *std, saf, fim, bcf* and *stf* genes are over-expressed in a *stpA* mutant after *Salmonella* growth until exponential phase [29]. The absence of the *pef* operon in the list of the StpA-dependent genes of this study could be due to the culture conditions used which were quite different from those considered optimal for Pef fimbriae expression [21]. In conclusion, in the culture conditions we used, i.e. bacterial culture at 37°C in TSB-MES pH 5.1 under standing conditions, the absence of Pef expression *in vitro* is mostly due to a strong silencing by H-NS on the PpefB promoter. StpA and Hha/YdgT also repress *pef* transcription at PpefB promoter. Moreover, Pef expression is also modulated through a weak post-transcriptional mechanism involving the stabilizing effect of CsrA on the *pefACDorf5orf6* transcript. A model for *in vitro pef* regulation summarizing all the results obtained in this study as well as those described for the regulatory protein CsrA is presented Figure 8.

H-NS, StpA, Hha and YdgT silence genes by affecting transcription initiation, elongation and termination. Initiation is blocked by inhibiting the RNA polymerase binding to promoters and by blocking promoter escape [51]. H-NS and StpA form homodimers and interact together to form heterodimers. Both homodimers and heterodimers assemble in large multimers that interact with DNA. Both can also associate with Hha and YdgT [52]. Experimental evidence and a mechanistic model recently described indicate that Hha and YdgT act primarily through H-NS and StpA to modulate gene expression [37,53]. In this model, Hha directly interacts with the N-terminal dimerization domain of H-NS or StpA and interaction of positively charged residues of Hha with DNA facilitates the formation of repressive nucleoprotein-DNA complexes. Recently, Hha-YdgT have been shown to favor H-NS DNA-bridging activity, thanks to conformational changes of H-NS from a close to an open state [54]. On the contrary, few reports show that Hha binds to specific regulatory sequences independently of H-NS, although Hha has no DNA binding domain [38,39,55]. A few other papers report a Hha direct regulation but careful interpretation should be done since, in these studies, Hha protein purification was performed in wild-type *E. coli* and thus a co-purification of Hha with H-NS and/or StpA could have occurred [56,57]. Our results with the Δ*hns*Δ*hha*Δ*ydgT* triple mutant strongly suggest that Hha and YdgT act independently of H-NS to modulate *pef* expression when bacteria are grown statically at pH 5.1 since an increase of PefA expression and a higher PpefB transcriptional activity have been observed in the triple mutant compared to the Δ*hns* mutant in this culture condition. As Hha-YdgT do not complement the Δ*hns*Δ*hha*Δ*ydgT* triple mutant in β-galactosidase assays, this strongly suggest an indirect activity of Hha-YdgT in this mutant. As StpA represses *pef* operon, the most probable hypothesis is that StpA in conjunction with Hha and YdgT silences *pef* transcription in the *hns* mutant. We tried to test whether the higher expression of PefA in the Δ*hns*Δ*hha*Δ*ydgT* triple mutant compared to the *hns* mutant could be due to StpA silencing through Hha/YdgT in the Δ*hns* mutant by constructing a Δ*hns*Δ*stpA* mutant. However, we never succeeded in constructing a double Δ*hns*Δ*stpA* mutant while we had no problem to transduce a *hns::kan* mutation in an *stpA* mutant complemented with *stpA*. The Δ*hns*Δ*stpA* double mutation is thus most probably lethal in *Salmonella*. In *E. coli*, Δ*hns*Δ*stpA* double mutants have been studied but a severe growth defect has been reported [58–60]. Consequently, we cannot completely rule out the hypothesis that Hha-YdgT act indirectly through another unknown regulatory protein. A recent work identified 120 genes regulated by Hha independently of H-NS/StpA [39] among which two fimbrial genes belonging to *sth* and *stj* operons, but not the *pef* operon. However, bacteria were grown in neutral medium in this study and consequently not in conditions optimal for Pef expression. Further studies are thus necessary to understand the mechanisms used by Hha and YdgT to regulate *pef* operon transcription.

**Figure 9.**
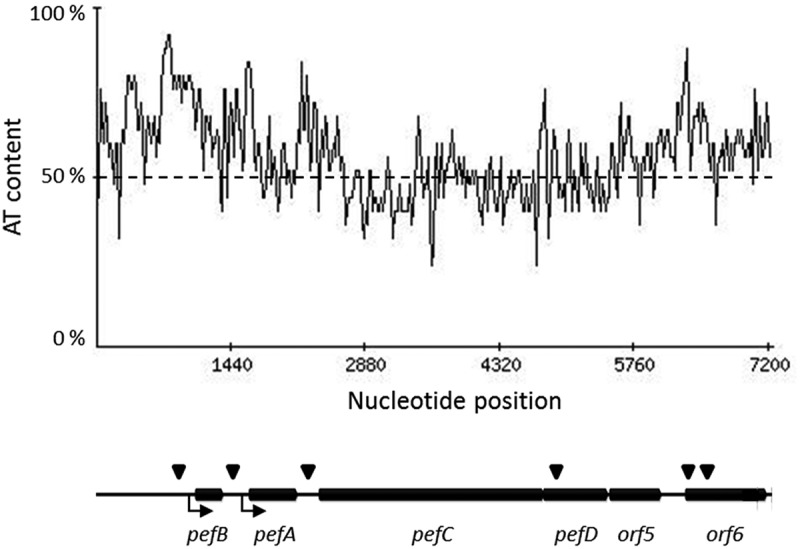
H-NS predicted sites and AT content of the *pef* operon. The upper part of the figure shows the AT percentage along the nucleotide sequence of the *pef* operon. A schematic diagram of the *pef* operon, with the H-NS predicted sites symbolized by black triangles, is represented in the lower part of the figure. The AT content was calculated using a window length of 25 bp. Potential H-NS binding sites in *pef* operon were searched using the Virtual Footprint software [72] and the H-NS position weight matrix from *E. coli* [61]. Only the sites with a score greater than 5.5 are indicated, while the maximum score for this matrix is 6.73. The experimentally identified PpefB and PpefA promoters are indicated by broken arrows.

While the precise mechanism of *pef* fimbriae regulation by Hha and YdgT remains to be determined, the silencing by H-NS most certainly involves a direct interaction of H-NS with the promoter regions upstream of *pefB* and *pefA* since a chromatin immunoprecipitation-on-chip analysis identified an H-NS binding site upstream of both ORFs [24]. Moreover, a search for sequences similar to the H-NS DNA binding motif determined in *E. coli* [61] allowed us to identify one putative H-NS high-affinity binding site in the promoter regions of both *pefB* and *pefA* (Figure 9). They are located approximately 100 bp upstream of the transcriptional start sites of both promoters. After binding of H-NS, the high AT richness of these regions, especially the one upstream of *pefB*, will then favor the oligomerization of H-NS along this DNA segment. Future work will include interaction and biophysical studies to verify our hypothesis concerning the ability of Hha-YdgT to regulate the PpefB promoter both through H-NS and/or StpA or independently of these NAPs according to the pH. Electrophoretic mobility shift assays, co-crystallization experiments with H-NS, StpA and/or Hha and their DNA target(s), and amino-acid mutagenesis will help clarify how Hha interacts with DNA according to the H-NS/StpA dependent or independent mechanism.

Adhesion to intestinal epithelium is an essential step for the colonization of the small intestine by *Salmonella* as bacteria must be in close contact with host cells to successfully invade them. Fimbrial adhesins represent one adhesion system responsible for this initial step of interaction between *Salmonella* and epithelial cells. As for most of *Salmonella* fimbriae, standard laboratory culture conditions do not allow the expression of Pef fimbriae. However, a study seeking for culture conditions able to induce Pef biosynthesis showed that maximal expression was obtained after growth of a *S*. Typhimurium wild-type strain at 37°C in rich acidic medium (LB broth buffered at pH 5.1 with MES) under standing culture conditions for 2 days [21]. In these conditions, Pef fimbriae were readily detected by immunofluorescence analysis in *S*. Typhimurium, as well as *pefA* and *pefB* transcripts. Moreover, PefA protein could be detected at a low level by flow cytometry in *S*. Typhimurium grown in similar conditions [8]. However, PefA subunit was not detectable by Western blot in *S*. Typhimurium wild-type strain cultivated in these conditions [7,22]. In agreement with these studies, we were unable to detect PefA by Western blotting in the wild-type strain whatever the growth conditions used. Our results with Δ*hns* and Δ*hha*Δ*ydgT* mutants also agree with the importance of standing cultures for the expression of Pef fimbriae. In contrast, in our hands, a rich medium buffered at pH 7.1 induced a stronger Pef expression than a rich acidic medium. Indeed, expression of PefA measured by Western blotting and transcriptional activity of the PpefB promoter was higher in Δ*hns*, Δ*hha*Δ*ydgT*, Δ*fimA-F*Δ*sirA* or Δ*stpA* mutants grown statically at 37°C in rich neutral medium (TSB broth buffered at pH 7.1 with MES) for 18 h. These contradictory results regarding optimum pH for Pef expression could be explained by different culture conditions. The main differences were the incubation time which lasted 2 days in the literature and 18 h in our experiments, and/or the presence of a pipette tip in the medium [21,22]. Another hypothesis would be that, in the wild-type strain, H-NS represses Pef expression more strongly at pH 7.1 than pH 5.1. More studies are required to decipher these expression discrepancies according to the culture conditions.

Among the many fimbrial adhesins of *S*. Typhimurium, the fimbriae Pef are involved in biofilm formation on chicken intestinal epithelium and adhesion to murine small intestine [6,17]. A glycomic study showed that Pef fimbriae specifically binds the Lewis X blood group antigen which is expressed mainly by crypt epithelial cells, suggesting a possible role for Pef fimbriae in adhesion of *Salmonella* to human crypt epithelium [62]. The growth conditions determined as optimum in our study for expression of PefA in Δ*hns*, Δ*stpA* or Δ*hha*Δ*ydgT* mutants can be considered as physiologically relevant since human gastrointestinal pH range from 6.6 in the jejunum to 7.5 in the ileum and oxygen level is low in the intestine [63,64]. Since most fimbrial operons of *S*. Typhimurium seem negatively regulated by one or several NAPs, this study could be extended to all the fimbrial operons in order to compare their mechanism of negative regulation. Finally, the identification of the transcriptional activator which relieves *in vivo* the silencing exerted by H-NS, StpA and Hha-YdgT would make it possible to better understand the role of the Pef fimbriae in animals.

## Experimental procedures

### Bacterial strains and culture conditions

Bacterial strains used in this study are listed in Table 1. *S*. Typhimurium 14028 is a virulent strain isolated from chicken tissues. *E. coli* MC1061 was used for cloning procedures. Strains were routinely grown in LB (10 g/L bactotryptone, 5 g/L yeast extract, 10 g/L NaCl) or TSB medium (BD Difco, No 211825) at 37°C with agitation. For *pef* operon transcription and PefA expression studies, *S*. Typhimurium strains were cultivated under standing or shaking conditions in 20 mL of TSB medium buffered to pH 5.1 or 7.1 with 100 mM MES [2-(N-morpholino)ethanesulfonic acid] and put in a 100 mL flask. When we used culture conditions similar to those of Nuccio et al. and Sterzenbach et al. [22,23], strains were grown in 5 mL TSB medium buffered to pH 5.1 or 7.1 with 100 mM MES in a 50 mL conical tube where a sterile 200 μL pipette tip was added. When necessary, antibiotics were added to the culture medium at the following concentrations: carbenicillin 100 μg/mL, kanamycin 50 μg/mL, chloramphenicol 34 μg/mL.

**Table 1.** Strains and plasmids used in this study.

Strain or plasmid	Relevant characteristic(s)^a^	Source or reference
Strains		
14028 14028Δ*fimA-F*Δ*sirA*	*S. enterica* subsp. *enterica* ser. Typhimurium wild-type strain 14028 isogenic mutant with the *fimA-F* operon and the *sirA* gene deleted	American Type Culture Collection This study
14028Δ*hns::kan*	14028 isogenic mutant with the *hns* gene deleted	[42] and this work
14028Δ*stpA::kan*	14028 isogenic mutant with the *stpA* gene deleted	This study
14028Δ*stpA*	14028 isogenic mutant with the *stpA* gene deleted	This study
14028Δ*hha*	14028 isogenic mutant with the *hha* gene deleted	This study
14028Δ*ydgT*	14028 isogenic mutant with the *ydgT* gene deleted	This study
14028Δ*hha*Δ*ydgT*	14028 isogenic mutant with the *hha* and *ydgT* genes deleted	This study
14028Δ*hns::kan*Δ*hha*Δ*ydgT*	14028 isogenic mutant with the *hns, hha* and *ydgT* genes deleted	This study
14028Δ*hns::kan*Δ*fimA-F*Δ*sirA*	14028 isogenic mutant with the *hns, fimA-F* and *sirA* genes deleted	This study
14028ΔPpefB	14028 isogenic mutant with the *pefB* promoter deleted	This study
14028Δ*hns::kan*ΔPpefB	14028 isogenic mutant with *hns* and the *pefB* promoter deleted	This study
14028Δ*stpA::kan*ΔPpefB	14028 isogenic mutant with *stpA* and the *pefB* promoter deleted	This study
14028Δ*hha*Δ*ydgT*ΔPpefB	14028 isogenic mutant with the *hha, ydgT* and the *pefB* promoter deleted	This study
MC1061	*E. coli hsdR mcrB araD*139 Δ(*araABC-leu)7679* Δ*lacX74 galU galK rpsL thi*	[69]
Plasmids		
pKD4	Vector carrying an FRT-Kan-FRT cassette (Cb^r^, Kan^r^)	[65]
pKD46	Carries λ-Red γ, β and *exo* genes under the control of P_ara_; temperature-sensitive replication (Cb^r^)	[65]
pCP20	Expression of FLP recombinase and temperature-sensitive replication (Cb^r^)	[70]
pACYC177	Cloning vector (Cb^r^, Kan^r^)	[71]
pAChns	pACYC177 containing *S*. Typhimurium 14028 *hns* gene and its RBS	[42]
pACstpA	pACYC177 containing *S*. Typhimurium 14028 *stpA* gene and its RBS	This study
pAChha-ydgT	pACYC177 containing *S*. Typhimurium 14028 *hha* and *ydgT* genes and their RBS	This study
pQF50Cm	Promoter-probe vector (Cm^r^)	[44]
pQF-PpefA	Transcriptional fusion PpefA-*lacZ* in pQF50Cm	This study
pQF-PpefB	Transcriptional fusion PpefB-*lacZ* in pQF50Cm	This study

^a^Cb^r^: carbenicillin resistance; Kan^r^: kanamycin resistance; Cm^r^: chloramphenicol resistance

### Cloning procedures and mutant construction

Plasmids used in this work are listed in Table 1. Promoters identified upstream of *pefB* or *pefA* were inserted upstream of promoterless *lacZ* gene in pQF50Cm in order to construct plasmid-based transcriptional fusions (Figure 3(a)). The fragments were amplified from *S*. Typhimurium 14028 genomic DNA using Herculase Hotstart DNA Polymerase (Agilent) and primers flanked by SphI and HindIII restriction sites at the 5ʹ and 3ʹ end respectively (Table S1). The resulting PCR products were digested with SphI and HindIII (Promega) and ligated into pQF50Cm cleaved by the same enzymes. All plasmids were validated by DNA sequencing.

For the construction of complementation plasmid pAChha-ydgT, *hha* and *ydgT* ORFs were amplified, including their ribosome binding sites (RBS), from *S*. Typhimurium 14028 genomic DNA using Herculase Hotstart DNA Polymerase (Agilent) and primers flanked by XhoI and ClaI restriction sites for *hha*, and SmaI and HindIII for *ydgT* (Table S1). First, the *hha* PCR product digested with XhoI and ClaI enzymes was ligated into pACYC177 cleaved by the same enzymes yielding the pAChha plasmid. Then, the *ydgT* PCR product digested with SmaI and HindIII was cloned into pAChha cleaved by the corresponding enzymes to generate pAChha-ydgT vector. The transcription of *hha* and *ydgT* in this recombinant plasmid is under the control of the promoter of the Kan^r^ gene of the pACYC177 vector which allows a constitutive expression of Hha and YdgT. In the same way, the complementation plasmid pACstpA was constructed by cloning *stpA* ORF, including its RBS, into pACYC177 cleaved by XhoI and HindIII. The sequence of pAChha-ydgT and pACstpA inserts were checked by DNA sequencing.

Chromosomal deletions were done in *S*. Typhimurium 14028 strain by the λ-Red recombinant method using primers stpA-P1/stpA-P2 (*stpA* deletion), hha-P1/hha-P2 (*hha* deletion), ydgT-P1/ydgT-P2 (*ydgT* deletion), sirA-P1/sirAP2 (*sirA* deletion), FimA-P1/FimF-P2 (*fim* deletion) as previously described [65] (Table S1). The same protocol was used to delete a 50 bp region encompassing the −10 and −35 boxes of the PpefB promoter. All deletion mutants were checked by PCR, using primers outside of the deletion site, and DNA sequencing. Construction of Δ*hns::kan*, Δ*hns::kan*Δ*stpA*, Δ*hns::kan*Δ*fimA-F*Δ*sirA*, and Δ*hns::kan*Δ*hha*Δ*ydgT* mutants was performed by transduction of the Δ*hns::kan* mutation in the wild-type, the Δ*stpA* mutant, the Δ*fimA-F*Δ*sirA* or the Δ*hha*Δ*ydgT* double mutants with a P22HT105*int* lysate as previously described [42]. Due to their genetic instability, all mutants containing an *hns* deletion were freshly prepared by transduction before each experiment and several mutants were studied in each experiment.

### Western blot analysis

Total bacterial extracts were prepared as previously described [66]. Bacterial proteins (an equivalent of 10^8^ bacteria/well) were separated by electrophoresis in 15% (wt/vol) SDS-polyacrylamide gel (PAGE) and transferred onto a nitrocellulose membrane. Immunoblotting were performed using either a mouse anti-Hsp60 monoclonal antibody (loading control; 1:6000; Enzo Life Sciences) or a rabbit anti-PefA polyclonal antibody (1:500) [8]. Horseradish peroxidase HRP-conjugated rabbit anti-mouse IgG (1:5000; Dako) or HRP-conjugated goat anti-rabbit IgG (1:5000; Dako) were used as secondary antibodies. Detection was performed by chemiluminescence with Fusion FX7 imaging system (Vilber Lourmat) using the SuperSignal™ West Dura Extended Duration Substrate (Thermo Scientific). Exposure times for PefA detection are indicated on all the blots. For Hsp60 detection, exposure times varied between 100 and 500 ms. At least two independent assays were performed for each experiment.

### Reverse transcription-PCR (RT-PCR)

Bacteria were harvested by centrifugation and bacterial RNA were stabilized by a 0.1% SDS, 1% acidic phenol, 19% ethanol mixture [67]. Total RNA were purified using the RNeasy Mini Kit (Qiagen) and genomic DNA was removed using the TURBO DNA-free kit (Ambion) according to manufacturers’ instructions. Then two micrograms of total RNA were reverse transcribed in presence of random hexamers using the AMV Reverse Transcriptase (Promega). PCR amplification mixtures contained 0.5 μM of each primer (Table S1), 200 μM deoxyribonucleoside triphosphate, 1.5 mM MgCl_2_, 1 U of GoTaq DNA polymerase (Promega) and 1X Green GoTaq Flexi Buffer (Promega). The temperature cycling for the amplification was performed in a Bio-Rad thermocycler (MyCycler) as follows: 1 cycle at 95°C for 3 min; 30 cycles at 95°C for 30 s, 60°C for 30 s and 72°C for 30 s; and finally, 1 cycle at 72°C for 5 min. Reactions without RT were performed to check the absence of DNA contamination in RNA samples. All RT-PCR experiments were duplicated from at least two independent bacterial cultures and RNA extractions.

### Measurements of β-galactosidase activity

The measures of the β-galactosidase activities were performed as described by Miller [68]. All assays were performed in triplicate from at least three independent bacterial cultures. β-galactosidase activities are expressed in Miller units.
