# Supplementary material for: H-NS is the major repressor of Salmonella Typhimurium Pef fimbriae expression
Source: Virulence. 2019 Oct 29;10(1):849–67. doi: 10.1080/21505594.2019.1682752 (PMC6844306; doi:10.1080/21505594.2019.1682752)
Supplement: Supplemental Material [file kvir-10-01-1682752-s001.docx]

**Table S1.** Oligonucleotides used in this study.

| Primer name | Sequence (5’-3’)^a^ | Description |
| --- | --- | --- |
| hha-P1 | CAATCATAGGTAGAATTTATGTCTGATAAACCATTAACTAAAACTGATTA**GTGTAGGCTGGAGCTGCTTC** | Construction of *hha* mutant |
| hha-P2 | ACCTGCGTGTTCTCTAAAAAGTAATGTAGCGTGATTAACGAATGAATTTC**CATATGAATATCCTCCTTAG** | Construction of *hha* mutant |
| ydgT-P1 | TCAGTGACTACTCCGTTGGCATTATATTTAATATGGATCAACTTTATATG**GTGTAGGCTGGAGCTGCTTC** | Construction of *ydgT* mutant |
| ydgT-P2 | GGCAAATATTATAAGGTTTTTGATGTTAAACGCTACTTTCTTTATTGCAC**CATATGAATATCCTCCTTAG** | Construction of *ydgT* mutant |
| fimA-P1 | ATTTATTACCGTGACGAAATGTCATATTCGCAAAGATTAATTACTGCGTC**GTGTAGGCTGGAGCTGCTTC** | Construction of *fimA-F* mutant |
| FimF-P2 | GCTAAGGCGATATAGTTCGCATAGAATTTCAGGGTAGCATTGCCGTTGTT**CATATGAATATCCTCCTTAG** | Construction of *fimA-F* mutant |
| SirA-P1 | TAAGGACGATTAACTATCAGTAGCGTTATCCCTATTCTGGAGATATTCCT**GTGTAGGCTGGAGCTGCTTC** | Construction of *sirA* mutant |
| SirA-P2 | AAAACGCCTTTGCGTCAAATATTTCACTCACTGGCTTGTTAACGTCTCCG**CATATGAATATCCTCCTTAG** | Construction of *sirA* mutant |
| stpA-P1 | AGTTTTTTGTTTTCTGCGTTAAAAGGTTTTTATTGATATGAATTTGATGT**GTGTAGGCTGGAGCTGCTTC** | Construction of *stpA* mutant |
| stpA-P2 | GACAGGAAACGAAGCGCCATCTGTTAAAAGCTATCCGTGATTAGATTAAG**CATATGAATATCCTCCTTAG** | Construction of *stpA* mutant |
| PpefBfw | TGTGGTGAAACATTCTGATTTATTTTGTTTGCATGGTAGAAAAGATCGCAT**GTGTAGGCTGGAGCTGCTTC** | Replacement of the *pefB* promoter with the kanamycin cassette |
| PpefBrev | ATCTCCGGCTCAATCTGGTGTTGTGACAGAAAATTTCCAAAGGAAACAAA**ATATGAATATCCTCCTTAG** | Replacement of the *pefB* promoter with the kanamycin cassette |
| pefBRI1050 | CTCGCATGCATACCATCCCAGCCTCGATCC | Cloning of PpefB region in pQF50Cm |
| pefBpQF | CTCAAGCTTCAGCGCCCCTCTTCTTATTCTGG | Cloning of PpefB region in pQF50Cm |
| pefB3'FWD | CTCGCATGCTTTCAGTACCAGCCTGAACC | Cloning of PpefA region in pQF50Cm |
| pefA5'REV | CTCAAGCTTCTTCATTGGCAGCGTGTGCA | Cloning of PpefA region in pQF50Cm |
| hhaXhoIfwd | CTCCTCGAGCAATCATAGGTAGAATTTATGTCTG | Cloning of *hha* in pACYC177 |
| hhaClaIrev | CTCATCGATGCGTGATTAACGAATGAATTTCC | Cloning of *hha* in pACYC177 |
| ydgTSmaIfwd | CTCCCCGGGGGGTGCAAAGAAAAGTCAAAAAC | Cloning of *ydgT* in pACYC177 |
| ydgTHindIIIrev | CTCAAGCTTCTTTCTTTATTGCACATAGCGCCAG | Cloning of *ydgT* in pACYC177 |
| stpAXhoI-FWD | CTCCTCGAGGTTAAAAGGTTTTTATTGATATGAATTTG | Cloning of *stpA* in pACYC177 |
| stpAHindIII-REV | CTCAAGCTTGCTATCCGTGATTAGATTAAGAAATC | Cloning of *stpA* in pACYC177 |
| pefB-FWD | TGCTGGCCAGAATAAGAAGAGG | RT-PCR on *pefB* gene |
| pefB-REV | GCTGGTACTGAAATACCCGTTG | RT-PCR on *pefB* gene |
| pefA-FWD | TCAGCCGAACCAGGTTGTTCAG | RT-PCR on *pefA* gene |
| pefA-REV | TGCTGGCGTTAGCGTTTACAGC | RT-PCR on *pefA* gene |
| pefC-FWD | CAGTGCCTATGCAAATGCCGAC | RT-PCR on *pefC* gene |
| pefC-REV | TGTTACTGTTGGAGCGCAGTGC | RT-PCR on *pefC* gene |
| orf5-FWD | CACGTTAACCAACCCGCAACAG | RT-PCR on *orf5* gene |
| orf5-REV | CCGCATAATGACTACGCTGCTG | RT-PCR on *orf5* gene |

^a^ Restriction sites are underlined. Sequences in bold are complementary to template plasmid pKD4.
